# Early- to mid-term outcome of a short, cementless, titanium, flat, tapered stem for primary total hip arthroplasty: an independent series

**DOI:** 10.1177/11207000231216421

**Published:** 2023-12-11

**Authors:** Jonathan Bourget-Murray, Brook Biniam, Raman S Bhullar, Paul Kim, Wade Gofton, Paul E Beaulé, George Grammatopoulos

**Affiliations:** Department of Surgery, Division of Orthopaedic Surgery, The Ottawa Hospital, University of Ottawa, Ottawa, Ontario, Canada

**Keywords:** Hip arthroplasty, microplasty, short stem, survival, Taperloc

## Abstract

**Introduction:**

This study aims to: (1) describe perioperative complications amongst patients who underwent primary total hip arthroplasty (THA) using a short cementless, titanium, flat, tapered stem; (2) estimate this stem’s early- to mid-term survival; (3) identify factors associated with revision arthroplasty; and (4) describe femoral remodelling at minimum 6 years postoperatively.

**Methods:**

A retrospective review of consecutive patients who underwent THA using a Taperloc Microplasty stem (Zimmer-Biomet, Warsaw, Indiana, USA) with minimum 2-year follow-up was performed. Surgeries were performed by 1 of 6, non-designer, arthroplasty surgeons between 2014 and 2018. Outcomes included perioperative complications including revision arthroplasty, and survival. Cox analysis was used to analyse the effect of different factors on risk of revision arthroplasty. Radiographs with 6-year follow-up served to describe femoral remodelling.

**Results:**

In 1205 patients, followed for 5.1 ± 1.4 years, the incidence of perioperative complication was 5.2% for which 29 patients (2.4%) required revision arthroplasty. The 5- and 7-year survival rates were 97.8% (95% CI, 96.9–98.5) and 97.0% (95% CI, 95.6–98.0), respectively. The only factor associated with revision arthroplasty was proximal femur morphology, as per Dorr classification (HR 1.24 [95%CI, 1.09–1.41]; *p* = 0.005). During radiographic assessment, 12% of patients showed ⩾25% of relative change in cortical thickness in Gruen zones 3 or 5. We observed calcar remodelling in 50% of radiographs while 10% showed presence of a pedestal sign.

**Conclusions:**

The 7-year survivorship of the Taperloc Microplasty stem is within National Institute for Health and Care Excellence (NICE) guidelines. Patients ⩽65 years with osteoarthritis and Dorr A/B femoral morphology may be ideal candidates for THA with this stem. Femoral remodelling is common and not associated with adverse outcome.

## Introduction

Total hip arthroplasty (THA) is extremely successful at alleviating pain and disability, providing substantial improvement in quality of life to patients.^1^ Given the projected rise in demand for THA, clinical strategies are needed to improve efficiency while maintaining quality, safety, and value of care for patients.

The use of less invasive surgical techniques is 1 feature of care that impacts patient recovery and accelerates rehabilitation. To facilitate femoral instrumentation and introduction of the prosthesis into the canal through these approaches, shorter femoral stems have been designed.^2,3^ The metaphyseal-based fixation of these short cementless stems was designed to preserve proximal bone density by reproducing physical loading to the proximal femur, thereby decreasing stress shielding.^4,5^ 1 such prosthesis is the Taperloc Microplasty Complete Hip System (Zimmer-Biomet, Warsaw, IN, USA) - a short cementless, titanium, flat, tapered stem. Despite sharing the same proximal geometry as the standard-length Taperloc Complete, which has an excellent long-term track-record and a current ODEP rating of 15A*,^6^ it is unclear how the shorter stem impacts performance metrics, survival, and proximal femoral remodelling over time. The few studies that have been published on this stem suffer from methodological limitations: single-surgeon (designer) case-series or have small cohort size.^7–10^ Given the increasing utilisation of less invasive techniques in combination with short cementless femoral stems, large multi-surgeon (non-designer) case-series are warranted.

The aims of this study were to: (1) describe perioperative complications amongst patients who underwent primary total hip arthroplasty (THA) using the Taperloc Microplasty stem; (2) estimate 5- and 7-year survival; (3) identify factors associated with revision arthroplasty; and (4) describe radiographic features of proximal femoral remodelling at a minimum 6 years postoperatively.

## Methods

### Study design, study participants, and data collection

This is an IRB-approved retrospective review of prospectively collected data from patients who underwent a primary, elective THA using the Taperloc Microplasty stem at a single academic, tertiary-referral hospital. All surgeries were performed by 1 of 6, non-designer, high-volume fellowship-trained arthroplasty surgeons between September 2014 and October 2018. A local arthroplasty database was queried to identify consecutive patients who underwent unilateral THA with a Taperloc Microplasty stem and who had a minimum 2-year follow-up. For the purpose of this study, patients who underwent simultaneous bilateral THA were excluded. 1205 consecutive Taperloc Microplasty stems were implanted in 1205 patients at our institution during the study period and served as the study cohort.

### Surgical procedures

All patients were treated in accordance with current local standard care pathways at the time of surgery. All patients received a weight-based dose of antibiotics and 1g of intravenous tranexamic acid prior to skin incision. Surgeries were performed in accordance with each surgeon’s preferred surgical technique: anterior approach (1330 hips, 94%), posterior approach (63 hips, 5.2%), and direct lateral approach (9 hips, 0.75%). The anterior approach to the hip were performed on either a standard operating table or an orthopaedic positioning table (Hana Orthopaedic Surgery Table, Mizuho OSI, Union City, CA) as per surgeon preference. Our institution’s experience with the anterior approach has been previously described.^11^ It is common practice at our centre for patients to be reviewed clinically at 2 weeks, 6 weeks, 6 months, 12 months and annually thereafter.

### Stem characteristics

The Taperloc Microplasty stem is a short cementless, collarless titanium alloy stem with a flat, tapered-wedge geometry design (3° bi-planar taper). The stem is coated proximally with a titanium alloy plasma spray coating for metaphyseal fixation. Trunnion offset options for this particular implant includes: (1) standard offset (133°); (2) high offset; or (3) XR 123° offset. It features a distal stem that is 35-mm shorter than the standard-length Taperloc Complete stem, which serves to enhances implant fit in femoral canals with a proximal/distal mismatch. The distal tip is rounded laterally to avoid varus positioning.

### Perioperative outcomes and survival

All patients’ Electronic Medical Records (EMR) were reviewed to identify perioperative complications including periprosthetic fracture (PPF); intra- and postoperative fractures, dislocation, aseptic loosening, painful impingement and periprosthetic joint infection (PJI).

Implant survivorship for the whole cohort was estimated at 5 and 7 years with revision for any reason as the endpoint. Date of last clinical follow-up was date of last clinical review. For patients who required a revision arthroplasty, indication and date of surgery were recorded and would serve as the date of last follow-up. Similarly, for patients who died, date of death as recorded in our EMR system served as the date of last follow-up.

Different patient-, surgical- and implant-related factors were tested for association with implant survivorship. Patient-related factors included age at index arthroplasty (categorised as ⩽65 or >65 years), sex, body mass index (BMI) <40 kg/m^2^ or ⩾40 kg/m^2^), laterality, and Dorr classification.^12^ Surgery-related factors included surgeon, indication for THA, and surgical approach. Implant-related factors include stem size, stem offset, femoral head size, and bearing surface.

### Radiographic assessments

2 fellowship-trained arthroplasty surgeons (JBM and GG) reviewed radiographs and performed all measurements. 100 radiographs from patients at a minimum 6-year follow-up were reviewed to describe proximal femur remodelling. The 2-week postoperative radiographs served as the baseline for comparison. The radiographic parameters reviewed included: (1) coronal alignment (varus or valgus position), defined as a coronal deviation of >3° from the longitudinal axis of the femur; (2) distal femoral cortical hypertrophy, assessed in Gruen zones 3 and 5. The relative change in cortical thickness (%) was measured and considered significant if increased ⩾25%. This technique has previously been described by way of normalising the differences in baseline cortical diameter;^13^ (3) subsidence (⩾ 5 mm), defined as the distance from the tip of the greater trochanter to the lateral shoulder of the femoral stem; (4) presence of cortical calcar rounding or atrophy, and; 5) presence of an intramedullary pedestal sign distal to the tip of the stem.

The latest clinical notes for patients with ⩾25% relative change in distal femoral cortical hypertrophy or an intramedullary bone pedestal were reviewed to determine if these changes were associated with adverse clinical significance, specifically thigh pain.

### Statistical analysis

Statistical analysis was performed using SPSS (version 26.0. IBM Corp.). We summarised demographic data and outcomes using descriptive statistics. These are presented as means with their corresponding standard deviations (SD). Categorical variables are presented as absolute numbers and percentages. Survival analysis, taking into account time to revision, was performed using Kaplan-Meier survival analysis with 95% confidence intervals (CI). Survivorship curves were tested using the Log Rank Test. Student’s *t*-test or chi-square test were used to assess the association between potential factors that might have influenced survivorship. Univariate analysis was performed to identify factors associated with revision arthroplasty. Factors with a trend to significance (*p* < 0.200) were included. Cox’s proportional hazards analysis was used to estimate the independent effect of each factor in multiple regression models. A *p*-value < 0.05 was considered significant.

## Results

The cohort consisted of 1,205 patients with a mean age of 63.5 ± 12.6 years followed for a mean of 5.1 ± 1.4 years (range 0.01 [early failure]–7.8). Cohort demographics and perioperative characteristics are presented in Table 1.

**Table 1.** Patient demographics and perioperative characteristics.

	Entire cohort (*n* = 1,205)	Revised (*n* = 29)	Non-revised (*n* = 1176)	*p*-Value
Age, years (SD)	63.5 ± 12.6	68.9 ± 11.1	63.4 ± 12.6	0.013^b^
Sex, *n* (%)				
Male	524 (43.5)	13 (44.8)	511 (43.4)	0.88^a^
Female	681 (56.5)	16 (55.2)	665 (56.6)	
BMI, kg/m^2^	28.0 ± 6.0	29.1 ± 6.2	28.0 ± 6.0	0.362^b^
Mean follow-up, years (SD)	5.11 ± 1.5	1.7 ± 2.0	5.2 ± 1.4	<0.001^b^
Dorr classification, *n* (%)				
A	866 (71.9)	18 (62.1)	848 (72.1)	0.111^a^
B	331 (27.5)	10 (34.5)	321 (27.3)	
C	8 (0.6)	1 (3.4)	7 (0.6)	
Laterality, *n* (%)				
Left	543 (45.1)	9 (31.0)	534 (45.4)	0.124^a^
Right	662 (54.9)	20 (69.0)	642 (54.6)	
Surgical approach, *n* (%)				
Anterior	1186 (98.4)	28 (96.6)	1158 (98.5)	0.413^a^
Posterior or lateral	19 (1.6)	1 (3.4)	18 (1.5)	
Indication for arthroplasty, *n* (%)				
Osteoarthritis	1179 (97.8)	27 (93.1)	1152 (98.0)	0.075^a^
Hip fracture	26 (2.2)	2 (0.2)	24 (2.0)	
Stem size, median (range)	12 (4–22)	12 (7–18)	12 (4–22)	0.258^b^
Stem offset, *n* (%)				
Standard	997 (82.7)	22 (75.9)	975 (80.9)	0.321^a^
High	208 (17.3)	7 (24.1)	201 (17.1)	
Mean head size, *n* (%)				
28	24 (2.0)	0 (0)	24 (2.1)	0.501^b^
32	618 (51.3)	14 (48.3)	604 (51.3)	
36	563 (46.7)	15 (51.7)	548 (46.6)	
Bearing surface, *n* (%)				
Metal-on-polyethylene	950 (78.8)	25 (86.2)	925 (78.7)	0.325^b^
Ceramic-on-polyethylene	255 (21.2)	4 (13.8)	251 (21.3)	

SD, standard deviation; BMI, body mass index.

a Chi-square statistical test.

b Student’s *t*-test.

The overall rate of perioperative complication was 5.2% (Table 2). At latest follow-up, 32 patients (2.65%) had died from causes unrelated to the index arthroplasty. Two of these patients had undergone revision arthroplasty for PPF. A total of 29 patients (2.4%) were revised. Indication for revision arthroplasty included PPF (14 hips, 1.16%), aseptic loosening (9 hips, 0.75%), PJI (6 hips, 0.5%), and painful impingement (1 hip, 0.08%). On average, revision arthroplasty took place 1.73 ± 1.95 years following index arthroplasty. Patients requiring revision arthroplasty were on average older (68.9 ± 11.1 years vs. 63.4 ± 12.6 years; *p* = 0.013). There were 16 intraoperative fractures; 6 fractures of the greater trochanter and 10 of the medial calcar.

**Table 2.** Perioperative complications.

Complication, *n* (%)	Cohort (*n* = 1,205)	Revised (*n* = 29)
Fracture	39 (3.2%)	14 (48.3%)
Intraoperative	16	
Greater trochanter	6	
Medial calcar	10	
Postoperative	23	14
Vancouver A	6	2
Vancouver B1	10	5
Vancouver B2	6	6
Vancouver B3	1	1
Dislocation	8 (0.7%)	0
Aseptic loosening	9 (0.7%)	9 (31.0%)
Painful impingement	1 (0.08%)	1 (3.4%)
Infection	6 (0.6%)	6 (20.7%)

Non-revised cases were treated either operatively or nonoperatively as deemed appropriate.

### Survival and risk factors for revision arthroplasty

**Figure 1.**
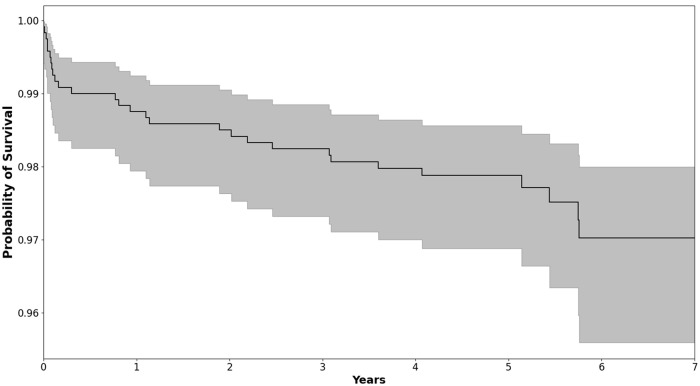
Kaplan-Meier survival curve for the Taperloc Microplasty Complete Hip System (Zimmer-Biomet, Warsaw, IN, USA) for the whole cohort, showing survival with revision for any reason as the endpoint with the 95% confidence intervals.

The overall 5- and 7-year survival for the Taperloc Microplasty was estimated to be 97.8% (95% CI, 96.9–98.5) and 97.0% (95% CI, 95.6–98.0), respectively (Table 3) (Figure 1).

**Table 3.** 5- and 7-year survivorship of the Taperloc Microplasty stem.

	Survivorship	95% CI	No. of patients at risk
5-year survivorship	97.8%	96.9 to 98.5	634
7-year survivorship	97.0%	95.6 to 98.0	143

CI, confidence interval.

There was no difference in survival between males and females (98.1% vs. 97.6%; log rank = 0.86). Patients ⩽65 years showed improved survival compared to patients >65 years (98.9% vs. 96.8%, log rank = 0.01). An association between survival and proximal femur morphology was detected (Dorr A, 97.1%; Dorr B, 92.4%; Dorr C, 87.5%; log rank = 0.057). Osteoarthritis, as an indication for arthroplasty, showed superior survival compared to fracture (98.0% vs. 92.0%; log rank = 0.034). In light of this, the estimated 5-year survivorship for the Taperloc Microplasty in patients ⩽65 years of age, treated for osteoarthritis, with Dorr A or B proximal femoral morphology is 98.1% (95% CI, 95.9–99.1). Applying Cox’s proportional hazards model revealed that only proximal femur morphology, as categorised by the Dorr classification was associated with increased risk of revision arthroplasty (hazard ratio [HR] 1.24; 95% CI, 1.09–1.41; *p* = 0.005) (Table 4).

**Table 4.** Cox’s proportional hazard analysis.

		Concordance = 0.71	
	Coefficient	95% CI	*p*-Value
Age	1.11	1.00–1.25	0.07
Indication for arthroplasty	1.15	0.78–1.73	0.48
Dorr classification	1.24	1.09–1.41	0.005

Diagnosis, osteoarthritis or fracture; Age, ⩽65 years or >65 years; Dorr classification, type A or type B or type C.

### Radiographic assessment

**Figure 2.**
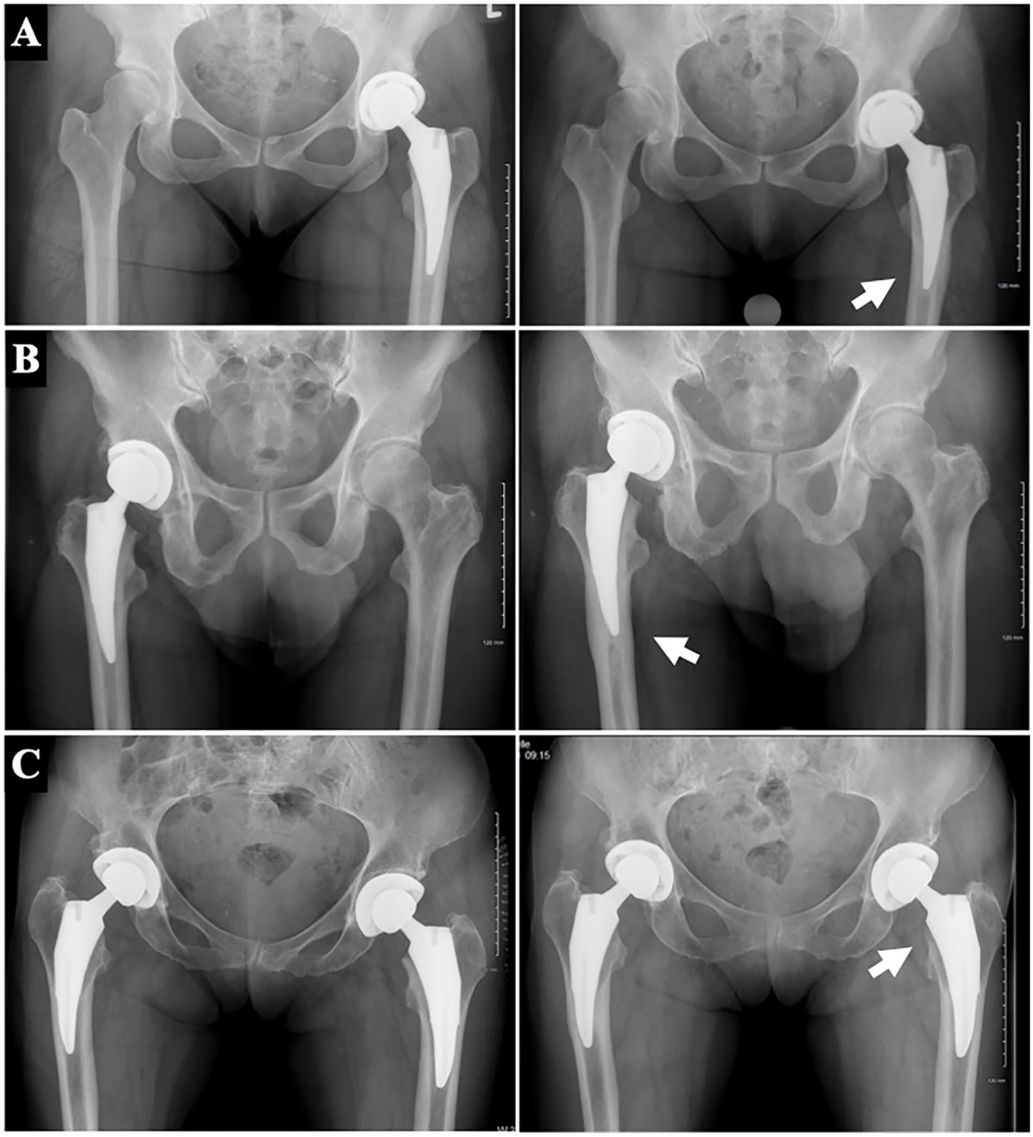
Clinically representative radiographs of proximal femoral remodelling at a minimum 6 years postoperatively. (a and b) are anteroposterior pelvis radiographs illustrating 2 relevant cases of ⩾25% relative change in distal femoral cortical hypertrophy in Gruen zone 3. Radiographs on the left are those taken 2-week postoperative and served as the baseline for comparison. (c) Anteroposterior pelvis radiographs illustrating medial calcar remodelling (rounding and atrophy).

Complete radiographic assessment is presented in Table 5. 8 femoral stems were placed in varus. 2 hips showed subsidence of ⩾5 mm. On average, cortical thickness increased by 9.8% in Gruen zone 3 and 9.1% in Gruen zone 5 at a minimum 6-year follow-up. Overall, 12% and 13% of radiographs demonstrated ⩾25% of cortical thickness in Gruen zones 3 and 5, respectively (Figure 2(a) and (b)). Some degree of medial calcar remodelling (rounding or atrophy) was appreciated in 50% of radiographs (Figure 2(c)). 10% of radiographs showed presence of a pedestal sign at the tip of the stem.

**Table 5.** Radiographic assessment.

	*n* = 100
Malposition (>3°)	8%
Subsidence (⩾5 mm)	2%
Mean cortical hypertrophy Gruen zone 3	9.8%
⩾25% of relative change in cortical thickness in Gruen zones 3	12%
Mean cortical hypertrophy Gruen zone 5	9.1%
⩾25% of relative change in cortical thickness in Gruen zones 5	13%
Calcar remodelling (rounding or atrophy)	50%
Intramedullary bone pedestal	10%

27 patients were identified to have either ⩾25% relative change in distal femoral cortical hypertrophy in Gruen zone 3 and/or 5 or a pedestal sign. Upon review of latest clinical encounter, 25/27 patients (92.6%) reported doing well, and denied any hip or thigh pain, the remaining 2 voiced non-specific hip pain but denied any thigh pain.

## Discussion

At a mean follow-up of 5.1 ± 1.4 years, this study showed an incidence of perioperative complications of 5.2% in which 2.4% of patients required revision arthroplasty. The 5- and 7-year survival were 97.8% (95% CI, 96.9–98.5) and 97.0% (95% CI, 95.6–98.0), respectively. The only factor associated with revision arthroplasty was proximal femur morphology, as per Dorr classification. The study also highlights that proximal femoral remodelling is common and is not associated with adverse outcome.

By way of addressing the practical challenges that surgeons face with utilising less invasive surgical techniques for THA, manufacturers have designed instruments and prostheses to improve surgical efficiency and facilitate introduction of the femoral stem into the canal.^2,3^ While the Taperloc Microplasty was designed with the same proximal geometry as the standard-length Taperloc Complete, it is unclear how changing length and geometry impacts the quality, safety, and survival of the implant. This study represents the largest independent series to report early- and mid-term outcome of the Taperloc Microplasty stem. This was possible given that at our institution, the anterior approach to the hip and the use of the Taperloc Microplasty has become the preferred surgical strategy for most elective primary THA since 2014.

The reported 5- and 7-year survivorship of the Taperloc Microplasty stem exceeded 97%, which is within National Institute for Health and Care Excellence (NICE) benchmarks.^14^ The stem performed particularly well in patients ⩽65 years, treated for osteoarthritis, with Dorr A or B proximal femoral morphology. However, this study also identified that 5.2% of patients experience a complication, with 2.4% requiring revision arthroplasty at a mean 5.1 years postoperatively. The most common indication for revision arthroplasty was PPF (14/29). This is clinically important since a PPF of the femur is a devastating complication with an associated 1-year mortality rate between 11% and 13%.^15,16^ Lamb et al.^17^ recently showed that collarless stem was an implant-related risk factor associated with an increase relative risk of PPF in the early postoperative period. The authors validated this finding using an *in vitro* biomechanical model, showing that a collar consistently improved the stability and resistance to fracture – whereby a PPF with a collarless stem occurs with less force than an otherwise identical collared stem. The authors theorised that during rotational injury, a collar may cause compressive loads to impart on the calcar, thus increasing the force required to cause a fracture. These findings are in keeping with those of Johnson et al.^18^ Given the increasing utilisation of short cementless femoral stems, future studies must clarify the effect of a collared short stems, such as the Taperloc Microplasty, on the risk of early periprosthetic femoral fracture.

Abdel et al.^19^ previously reported that female sex and age (>65 years) were factors that carry an increased risk of intraoperative fracture. While our univariate survival analysis did show improved survival in patients ⩽65 years, our Cox proportional hazards model did not identify age or sex as being associated with increased risk of revision arthroplasty. It did, however, identify proximal femur morphology, as categorised by the Dorr classification, to be associated with increased risk of revision arthroplasty. This is in keeping with recent work from McGoldrick et al.^20^ who illustrated that a canal flare index (CFI) >3.17 can serve as a predictor of early fracture when using short, collarless stems. Whether surgical approach is associated with PPF remains unclear; there is some evidence to suggest an increased risk of PPF during an anterior approach THA.^21^ Together, these findings highlight the need for caution in using a short, cementless, collarless stem for and anterior approach THA in certain patients. It is the authors’ opinion that choice of approach should be guided by surgeon experience (steep learning curve associated with the anterior approach) and patient factors (i.e., pelvic and proximal femur morphology, body habitus). Perhaps cautious use of the Taperloc Microplasty stem is warranted in patients >65 years of age, especially those with proximal femoral morphology categorised as Dorr C.

Our study expands on current knowledge from similar, yet smaller cohort studies investigating the use of the Taperloc Microplasty (Table 6). Saragaglia and Orfeuvre^10^ reported a survivorship of 100% at a mean 61-month follow-up. However, their cohort consisted of only 103 patients operated on by a single surgeon and had significant exclusion criteria. Meanwhile, Molli et al.^3^ previously compared patients who received a Taperloc Microplasty to patients who received a standard-length tapered titanium porous plasma-sprayed femoral stem (Mallory-Head Porous [MHP]; Biomet, Inc, Warsaw, IN, USA). The authors showed that a higher intraoperative fracture rate occurred with the standard-length stem (*n* = 12; 3.1%) compared with the short stem (*n* = 1; 0.4%). The authors observed an overall incidence of stem failure of 0.26% in the standard-length stem group and 0.37% in the short-stem group at a mean follow-up of 29.2 months.^3^ Since traditional stem designs have been successful with survivorship exceeding 95% at 10 years, it will be paramount to compare the 10-year survival of the Taperloc Microplasty when these become available.

**Table 6.** Clinical studies investigating the use of the Taperloc Microplasty stem.

Author/year	Aim of study	Study characteristics	Outcomes
Molli et al.^3^ (2012)	This study compares perioperative complications, short-term survivorship, and pain and function scores between patients who received an MHP femoral component or a Taperloc Microplasty	• 606 patients (658 hips) • 2 surgeons • Minimum follow-up: 0.8 months	Higher rate of intra-operative complications with the standard-length stems (3.1%; 3 trochanteric avulsions, 9 femoral fractures) compared with the shorter stems (0.4%; 1 femoral fracture). There were no differences in implant survival, HHA, and LEAS score between groups.
Tamaki et al.^22^ (2018)	Identify factors predicting risk of PPF during DAA THA	• 851 patients • 4 surgeons • Minimum follow-up: 3 months	17 PPF (2.0%) were observed, including 10 intraoperative (1.2%) and 7 postoperative (0.8%) fractures. The occurrence rate of fractures using the Taperloc Microplasty was significantly higher compared with that using standard stems (*p* = 0.02).
Lombardi et al.^7^ (2018)	Single centre’s early experience using the Taperloc Microplasty	• 92 patients (93 hips) • Mean follow-up: 4.5 years (2-6)	HHS improved from 52.5 to 84.7. 1 stem was revised. 2 patients required revision surgery. Radiographic assessment demonstrated no evidence of loosening, osteolysis, distal hypertrophy, or pedestal formation.
Nahas et al.^8^ (2018)	(1) Determine stem revision rate, (2) Describe complications, Oxford hip score, and (3) radiographic evidence of lucency	• 196 hips • Single surgeon • Mean follow-up: 36 months (5-75)	(1) One patient required revision surgery. (2) 2 dislocations and 1 stem subsidence. Oxford hip scores increased on average from 21 to 45. (3) There were no signs of radiographic loosening.
Gallart et al.^9^ (2019)	(1) evaluate clinical and radiological outcomes and (2) evaluate mid-term survivorship	• 32 patients (40 hips) • Median follow-up: 36.5 months (26-68)	MAS improved from 11.5 to 17.5 (*p* < 0.05). Subsidence (⩾3 mm) was detected in 1 patient. Varus malalignment was observed in 4 patients. No stems were revised. No patient reported thigh pain.
Saragaglia and Orfeuvre.^10^ (2020)	(1) Determine survival of the Taperloc Microplasty, and (2) Have the expected benefits of short stems been confirmed?	• 103 patients (110 hips) • 1 surgeon • Mean follow-up: 61 months	3 dislocations (2.5%). At follow-up the MAS was 17.8 ± 0.8 and the Oxford score 13.1 ± 3.5. On x-rays, 9 pedestal signs (7.5%), 6 calcar atrophies (5%), no cortical hypertrophy >2 mm, no stem subsidence > 5 mm, and no radiolucent line. Stem survivorship at 61 months was 100% (95% CI: 0.905; 1.095).
Won et al.^23^ (2021)	Determine the difference between short- and standard-length stems in terms of, (1) frequency or severity of thigh pain; (2) HHS; (3) implant loosening; and (4) BMD.	• 75 patients • 3 surgeons • Minimum follow-up: 5 years	(1) No difference in thigh pain. (2) No difference in HHS. (3) No loosening or revisions. (4) Patients with Taperloc Microplasty showed a slightly smaller decrease in BMD in Gruen Zones 2, 3, and 5.
Crawford et al.^13^ (2021)	(1) Determine if DFCH is associated with thigh pain following DAA THA. (2) Determine if patient age, gender, activity level or stem size is associated with the development of DFCH.	• 718 patients (820 hips) • 3 surgeons. • Mean clinical follow-up: 3.2 years (1.3–6.2) • Mean radiographic follow-up: 2.9 years (1–7.9)	(1) 52 hips (18%) had ⩾25% DFCH in Gruen zone 3 and 91 hips (31%) in zone 5. DFCH was not associated with thigh pain. (2) Stem size was positively correlated with DFCH in zone 3 and inversely related in zone 5.

PPF, periprosthetic fracture; HHS, Harris Hip Score; LEAS, Lower Extremity Activity Scale score; DAA, direct anterior approach; MAS, Merle d’Aubigné score; BMD, bone mineral density; DFCH, distal femoral cortical hypertrophy.

This study further described femoral remodelling around the Taperloc Microplasty at a minimum 6-year follow-up. Notably, we show that on average cortical hypertrophy develops in Gruen zones 3 and 5. These findings corroborate those of Crawford et al.,^13^ who at a mean 3.2 years postoperatively showed that 74% of patients developed cortical hypertrophy in Gruen zone 3 and 56% of patients in Gruen zone 5. We also describe a high incidence of remodelling of the medial calcar (rounding or atrophy). Together, these findings are suggestive that the design of the Taperloc Microplasty may allow for some distal load transfer despite its proximal coating meant for metaphyseal fixation. While the association between femoral remodelling and functional outcome was not the primary purpose of this study, it did not appear to have clinical significance in our patient cohort.

The limitations of our study are those inherent to large administrative databases which include, but are not limited to, misclassification, and lack of completeness and correctness of the data.^24^ This study is also a retrospective review of prospectively collected data and suffers from all associated biases, including selection bias. In addition, we could only include patient and surgery variables available within the data storage. However, our study currently serves as the largest cohort investigating the outcomes and survivorship of patients undergoing an elective THA with the Taperloc Microplasty stem. Secondly, all surgeries were performed by high-volume arthroplasty surgeons who were beyond the learning curve and proficient with THA through the anterior approach.^11^ Therefore, the results of this study may not be comparable or generalisable to smaller volume centres or to less experienced surgeons that are at the beginning of their learning curve. Thirdly, our EMR captures any Emergency Department visits or hospital admission to any hospital facility in the region. This process allowed us, for the purpose of this study, to capture any patient that may have presented to a peripheral hospital with a complication or undergone a hip revision elsewhere. However, it is plausible that some patients may not have been accounted for should they have received care outside our EMR’s catchment area. Fourthly, our Cox model suffered from a fairly unbalanced dataset. Our study would have potentially benefited from combining our institutional data with data from other centres and creating different re-sampled datasets. This would have allowed us to build models that use all of the samples from the revised group and non-differing samples of the non-revised class, thereby creating a balanced dataset. Lastly, a variety of short cementless femoral stems have been designed and are on the market; thus, no suggestion can or should be made as to whether the findings of this study are generalisable to other short stems or whether they are specific and only applicable to the implant studied. Further longer-term studies are required to elucidate this and determine whether our observations may translate to other femoral component designs.

## Conclusion

This multi-surgeon study from an independent, high-volume academic centre illustrates the safety and efficacy in the use of the Taperloc Microplasty stem for primary THA, with an overall 7-year survivorship rate exceeding 97%. Younger patients (⩽ 65-years) with osteoarthritis and Dorr A or B proximal femoral morphology seem to be the ideal candidates for this short cementless, titanium, flat, tapered stem. Features of femoral remodelling are commonly appreciated at early to mid-term follow-up, but these do not appear to be associated with poor clinical outcomes.
